# The best practice for preparation of samples from FTA^®^cards for diagnosis of blood borne infections using African trypanosomes as a model system

**DOI:** 10.1186/1756-3305-4-68

**Published:** 2011-05-07

**Authors:** Heba A Ahmed, Ewan T MacLeod, Geoff Hide, Susan C Welburn, Kim Picozzi

**Affiliations:** 1 Centre for Infectious Diseases, Division of Pathway Medicine, School of Biomedical Sciences, College of Medicine and Veterinary Medicine, University of Edinburgh, 49 Little France Crescent, Edinburgh, EH16 4SB, UK; 2 Faculty of Veterinary Medicine, Zagazig University, 44611, Egypt; 3 Centre for Parasitology and Disease, School of Environment and Life Sciences, University of Salford, Salford, M5 4WT, UK

## Abstract

**Background:**

Diagnosis of blood borne infectious diseases relies primarily on the detection of the causative agent in the blood sample. Molecular techniques offer sensitive and specific tools for this although considerable difficulties exist when using these approaches in the field environment. In large scale epidemiological studies, FTA^®^cards are becoming increasingly popular for the rapid collection and archiving of a large number of samples. However, there are some difficulties in the downstream processing of these cards which is essential for the accurate diagnosis of infection. Here we describe recommendations for the best practice approach for sample processing from FTA^®^cards for the molecular diagnosis of trypanosomiasis using PCR.

**Results:**

A comparison of five techniques was made. Detection from directly applied whole blood was less sensitive (35.6%) than whole blood which was subsequently eluted from the cards using Chelex^®^100 (56.4%). Better apparent sensitivity was achieved when blood was lysed prior to application on the FTA cards (73.3%) although this was not significant. This did not improve with subsequent elution using Chelex^®^100 (73.3%) and was not significantly different from direct DNA extraction from blood in the field (68.3%).

**Conclusions:**

Based on these results, the degree of effort required for each of these techniques and the difficulty of DNA extraction under field conditions, we recommend that blood is transferred onto FTA cards whole followed by elution in Chelex^®^100 as the best approach.

## Background

Blood and buffy coat samples have been routinely collected on ordinary filter paper for analysis and detection of different blood pathogens [1-7]. Although collection of blood samples on filter paper for large scale sampling seems to be more convenient than other sampling procedures, such as *in situ *DNA extraction [2], filter papers are not suitable for long term storage, as they do not protect the sample from spoiling and degradation [8].

Flinders Technology Associates (FTA) technology has improved upon this paper based system. The FTA^®^card has been designed to fix and store nucleic acids directly from tissues, allowing the collection and archiving of nucleic acids [9,10]. The FTA matrix is impregnated with protein denaturants that cause lysis of cells and any organism on contact. Moreover, these chemicals inhibit detritivores during drying to ensure the safe handling of cards without risk of biohazards [9,11]. FTA technology also includes chelating agents and a free-radical trap designed to deal with atmospheric pollutants, thus protecting the entrapped nucleic acids for at least six years at room temperature [11].

FTA^®^cards have been used for blood storage [12,13], detecting bacterial pathogens [11,14], detection of plant genes [15], specific detection of protozoa in water [16], detection of viral genomes [10,17,18] and in forensic human biology [19]. The use of FTA^®^cards has been extended to include DNA detection from pathogenic protozoa and pathogenic organisms isolated from food and clinical specimens using PCR [20].

The feasibility of FTA^®^cards for the shipment, storage and detection of RNA rabies virus has recently been evaluated by Picard-Meyer *et al. *[10]. The authors concluded that the chemicals impregnated in the filter paper have made the samples no longer infectious; subsequently the samples do not induce any biohazards especially during shipment. Moreover, the use of FTA^®^cards facilitates the large scale field sampling of viruses infecting plants and yields DNA with sufficient stability, quantity and quality for further screening over time of storage at room temperature was reported [18]. The authors successfully extracted viral DNA from FTA^®^cards stored at room temperature for nine months.

In trypanosome diagnosis, FTA^®^cards have been used for the storage of many materials including blood, buffy coat and extracted DNA [21-26]. The use of FTA^®^cards for storage of midgut material originating from tsetse flies has been documented by Adams *et al. *[27,28]. They noticed lower trypanosome detection using PCR than was expected and suggested several reasons for this, including the sub-optimum binding of trypanosome DNA to the FTA^®^cards and the uneven binding of the trypanosome DNA throughout the card due to competition with midgut material. The uneven distribution was reported to be the main contributing factor to this decreased sensitivity and that this was compounded when examining infections with low parasite densities [29]. Although FTA^®^cards are a reliable medium for storage and transport of blood samples for PCR-based assays, long-term storage (>9 months) has been shown to prevent the complete removal of blood proteins from discs by washing [23].

The present study was aimed at investigating the best approach for the preparation of blood samples applied on FTA^®^cards for the molecular diagnosis of trypanosomes. This aim was achieved by comparing three sample preparations: whole blood applied directly onto FTA^®^cards, lysed blood on FTA^®^cards and DNA extracted directly in the field using commercial kits. Lysed blood, where the genetic material within the sample was released from the cellular membranes prior to FTA^®^card application, was examined as a possible solution to overcome the localization of DNA across the surface of the membrane. Additionally, the use of Chelex^®^100 resin (previously described by Becker *et al. *[23] for elution of trypanosome material from FTA cards) was investigated as a possible approach to elute DNA from preprepared FTA^®^cards. These five protocols were compared to establish the most successful method of detection of trypanosome infections from FTA^®^cards.

## Results

**Figure 1:**
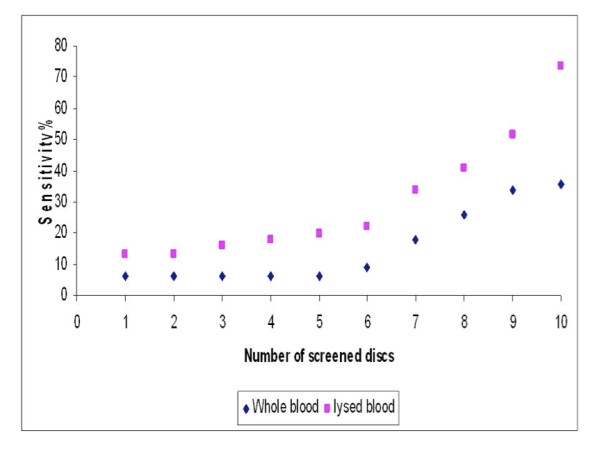
**Cumulative number of examined FTA discs containing whole and lysed blood in relation to the sensitivity of detecting the trypanosome DNA by PCR**.

Three different sample preparations were collected from 300 animals. These preparations included, whole blood applied on FTA^®^cards, lysed blood on FTA^®^cards and direct purification of DNA from blood in the field. Ten discs from each FTA^®^card applied blood sample were examined individually using TBR-PCR for the detection of *T. brucei *s.l. The sensitivity of the tests were calculated compared to the gold standard which was defined as the total number of PCR positive results from amplification of trypanosome DNA from samples applied to FTA^®^cards and/or from directly extracted trypanosome genomic DNA. Figure 1 shows that there is an increase in cumulative sensitivity as the number of individually examined discs was increased. The same phenomenon was observed from both whole and lysed blood samples.

For whole blood the sensitivity of PCR screening of up to five discs remained constant at 5.9%, compared to the gold standard, which increased to 35.6% when 10 separate discs were examined (Table 1). The sensitivity of detection of lysed blood samples increased from 12.9% by examining 1-2 discs to 73.3% by screening 10 discs when compared to the gold standard. Therefore the release of the genomic material from both the host and any infectious agent into a homogenous solution prior to application of the sample onto FTA^®^cards significantly improved the detection (p < 0.0001).

**Table 1:** Sensitivity of the used sample preparations compared to the gold standard for identifying *T. brucei *s.l. by PCR (n = 300)

Material	True positive	False positive	True negative	False negative	% NPV (95%CI)	% Sensitivity (95%CI)	Kappa value
Whole blood on FTA^®^cards (10 separate discs)	36	0	199	65	75.4% (70.2-80.6)	35.6% (26.3-45)	0.4

Whole blood on FTA^®^cards (elution)	57	0	199	44	81.9% (77.1-86.7)	56.4% (46.8-66.1)	0.6

DNA extract	69	0	199	32	86.1% (81.7-90.6)	68.3% (59.2-77.4)	0.7

Lysed blood on FTA^®^cards (elution)	74	0	199	27	88.1% (83.8-92.3)	73.3% (64.6-81.9)	0.8

Lysed blood on FTA^®^cards (10 separate discs)	74	0	199	27	88.1% (83.8-92.3)	73.3% (64.6-81.9)	0.8

However, although the lysis approach improved sensitivity, it is costly and time consuming to use due to the numerous individual PCR reactions per sample. In order to reduce these overheads, genomic material was eluted from 10 washed discs by collectively heating the preparation at 90°C in the presence of 60 μl of 5% (w/v) aqueous suspension of Chelex 100^® ^resin. Five microlitres of eluted DNA extract from each sample was examined by PCR in single reaction and the sensitivity of the different preparations were compared to the gold standard defined above for further evaluation of the lysis and elution protocols (Table 1).

The results show that the sensitivity of a single PCR reaction using 5 μl of DNA eluted with Chelex^®^100 solution from 10 discs containing whole blood significantly increased the sensitivity from 35.6% to 56.4% (Table 1) compared to non eluted whole blood taken from 10 separate discs (χ_1_^2 ^= 7.3, p = 0.007). A comparison of the sensitivity of detection of *T. brucei *s.l. DNA from lysed blood with Chelex^®^100 eluted samples from the lysed blood showed no difference (Table 1). A comparison of the sensitivity of using Chelex^®^100 eluates from whole and lysed blood showed an increase from 56.4% to 73.3% but this was not significant (χ_1_^2 ^= 1.7, p = 0.2). When comparing the PCR detection sensitivity of Chelex^®^100 eluates of whole blood (56.4%) and lysed blood (73.3%) with DNA extracted directly in the field (68.3%) there was no significant difference. Therefore, these two PCR detection protocols based on Chelex^®^100 elution are comparable in sensitivity to DNA prepared directly under field conditions at the point of collection.

Table 1 shows the Kappa values for the degree of agreement between the amplification of *T. brucei *s.l. using different preparation and processing methods compared to the gold standard. There was fair agreement between the detection of the DNA in whole blood samples using separate 10 discs for the PCR and the gold standard. Moderate agreement was seen when amplifying the parasite genomic material from eluates of whole blood discs, good agreement was observed in the amplification of DNA extracted in the field and very good agreement was found for both lysed blood samples. However, the lack of significant difference in sensitivity between the latter four processes suggests that each show equally good agreement with the gold standard.

## Discussion

FTA cards are considered reliable medium for the storage and transport of blood samples for molecular diagnosis of blood borne infectious diseases. The most important obstacle of processing the samples from the card matrix is the localized trapping of the genomic material after application of the sample. Moreover, long-term storage of the sample might hinder the release of the entrapped DNA from the card matrix for molecular diagnosis. This study aimed to investigate the application and processing of blood samples on the FTA matrix to improve the detection of trypanosomes by PCR.

The sensitivity of detecting positive discs containing whole blood was constant at 5.9% using one to five discs, after which the sensitivity increased to reach a maximum of 35.6% when all 10 discs were included (Figure 1). This was due to increasing the chance of obtaining parasite DNA in the examined disc as demonstrated previously by Cox *et al. *[29]. However, using discs containing lysed blood samples, the sensitivity of detecting positive discs using PCR increased to 73.3% using 10 discs. Comparing the sensitivity of examining one disc containing whole blood and lysed blood (5.9% and 12.9%, respectively), it was noticed that the lysis step doubled the sensitivity of the screening protocol; this was also the case when comparing the sensitivity of examining 10 discs from the two preparations using separate PCR reactions (35.6% and 73.3%, respectively).

The improvement in detection trypanosome target sequence was clear using the lysed blood samples; however, it required the screening of at least 10 discs to achieve a sufficient sensitivity when compared to the gold standard. The use of increased numbers of discs improved detection due to the increased chance of selecting a region of the card where parasitic DNA was stored. Although using a single disc screening protocol may be valid where infection intensities are generally very high, it may completely underestimate prevalence if the infection intensities are generally very low in the population [29]. Therefore, in order to decrease the probability of false negative results from using a single disc, examination of more discs would give more accurate estimation of the disease prevalence.

Despite the higher sensitivity of examining more discs, the use of 10 discs from whole blood or lysed blood samples with a separate PCR reaction on each disc was considered time consuming and expensive. Moreover, the use of 10 discs for detecting each trypanosome species would quickly use all the archived material on the card matrix. This was overcome by elution of DNA from FTA^®^cards containing either whole blood or lysed blood using 5% Chelex^®^100 resin aqueous suspension [23]. In both cases, this approach gave a sensitivity that was no different to the 10 disc approach using the TBR-PCR. The sensitivity of using lysed blood eluate in detecting trypanosome DNA was not significantly different than the PCR detection from DNA directly extracted in the field suggesting that these methods are as good and also more convenient alternatives.

Relating parasite density to PCR positivity on FTA cards can be problematic, however, Cox *et al. *[29] have recently suggested that the level of parasitaemia is related to the number of punches that are positive. Hence if 1/8 punches are positive the infection rate is 0.1 trypanosome per ml, 6/8 punches positive represents one trypanosome per ml and finally a 100% detection rate where there are greater than 10 parasites per ml. In the current work, however, sensitivity was increased by either elution of DNA or pre lysis of material before application to FTA card. This would suggest that parasite densities could be higher than those suggested by PCR from FTA cards. Although this would not affect the positivity status when parasitaemia was high it could have an affect when measuring density in low parasitaemias (particularly if trypanosomes clumped in particular areas of the card). Becker *et al. *[23] have published a quantitative real time method for the enumeration of trypanosomes from FTA card and this could provide an approach for measuring trypanosome populations from FTA samples. However, given the fact that trypanosome populations do fluctuate (due to antigenic variation) assessing if the animal is positive is more important in prevalence studies than the number of trypanosomes present in the animal.

Research has focused on the development of alternative extraction protocols that overcome the drawbacks of the conventional methods, but at the same time producing efficient DNA yield in both the quantity and quality to be easily amplified using PCR [30]. DNA elution from blood applied on FTA^®^cards or filter papers using 5% aqueous Chelex^®^100 solutions appear to be more convenient and cheaper than DNA extraction using extraction kits [23]. Other studies have shown that the benefits from using Chelex^®^100 to extract DNA from blood result in a six-fold increase in extraction efficiency [31] although in our study it only doubled the sensitivity. However, these approaches are better than conventional methods of DNA extraction that require large volumes of blood and the use of health hazard organic solvents [32]. A demonstration of the universal usefulness of Chelex^®^100 is that it has been used to elute the genomic DNA from a range of other parasites immobilized on filter paper including *Theileria parva *[30,33] and *Plasmodium falciparum *[34]. In the case of trypanosomes it has been used to elute from filter paper [1,6,35-38], buffy coat [2,38,39] and CSF [40]. Our study demonstrates the important application of it for use with FTA cards.

From the previous studies, it is clear that the use of Chelex^®^100 is effective in stabilizing the extraction of highly pure DNA from blood samples that are notorious for the carryover of PCR inhibitors [30]. The presence of Chelex overcomes the problems associated with inhibitory effects of the blood components such as heme, lactoferrin, IgG and non-target DNA [29,35]. Moreover, Chelex^®^100 protects DNA from the effects of high temperature that can cause degradation of DNA by chelating metal ions which might act as catalysts in the breakdown of DNA at high temperature in low ionic strength solutions [41].

DNA extraction from different samples including blood, CSF, tissues and cultures is an important step for conducting PCR for the diagnosis and research purposes. Extraction of DNA using different kits has been proven to recover high molecular weight DNA but this process is time consuming and expensive. Moreover, these methods require several steps and may include the transfer of DNA extracts to additional containers and columns increasing the chance for cross-transfer of samples or the introduction of contaminants [42]. ChargeSwitch^® ^gDNA kit used in this study to extract DNA from blood did not require the use of any electrical equipment because it depends on the use of magnetic beads, however, it was time consuming and expensive. For extracting DNA from one sample using ChargeSwitch^® ^gDNA kit, the cost was £4.20 compared to £2.70 and £3.20 (2010 prices) using whole blood and lysed blood on FTA^®^cards, respectively.

In conclusion, successful molecular diagnosis requires the availability of genomic material of an appropriate quality and concentration to be present within the sample under examination. The insignificant difference between using DNA eluted (by Chelex^®^100) from whole blood, lysed blood discs and DNA extracted using kits, suggests that the use of any of these aforementioned preparations are suitable for obtaining trypanosome genomic materials for molecular analysis. The choice will therefore be based on economic and time factors. Direct DNA extraction in the field using kits can be excluded due to the expensive kits and time taken for extracting DNA.

## Conclusions

The current work recommends that whole blood samples are collected using FTA^®^card technology with DNA being eluted using Chelex^®^100 once the investigator has returned to the laboratory. These steps should increase the sensitivity of the molecular diagnosis of trypanosome infections as it overcomes any problems associated with uneven distribution of parasite DNA on the card matrix.

## Methods

### Sample collection

In this study three sample preparations were collected under field conditions from 300 cattle from Apac and Lira districts, from Uganda during baseline sampling of the Stamp out Sleeping Sickness campaign in 2006 [43] These preparations included whole and lysed blood applied on FTA^®^cards and DNA extracted using ChargeSwitch^®^gDNA kits. The lysed blood was obtained by 1:1 dilution of the blood sample using sterile water, after allowing time for lysis to take place; the sample was then applied to the FTA matrix.

### Preparation of samples

For samples applied on FTA^®^cards, 0.2 mm discs were cut from the card for PCR analysis. The number of discs to be examined were cut and placed together into a micro-centrifuge tube for washing. To avoid cross contamination between samples, an equivalent number of discs were taken from a blank filter paper after each sample. The discs were washed twice, for 15 minutes each, in 200 μl (for each disc) of Whatman FTA purification reagent. The discs were then washed twice for 15 minutes in 200 μl (for each disc) 1 mM TE buffer (10 m M Tris-HCL pH 8.0; 1 mM EDTA pH 8.0), were then transferred to PCR tubes and left to dry at room temperature for at least 90 minutes [21]. Ten discs from both whole blood and lysed blood FTA^®^card preparations were examined by PCR (each disc examined with a separate PCR reaction).

The DNA from a further 10 prepared discs from each FTA^®^card sample was eluted collectively by heating the discs for 30 minutes at 90°C in 60 μl of 5% (w/v) aqueous suspension of Chelex 100^® ^resin (sodium form, 50-100 dry mesh, Sigma) [23]. For PCR, 5 μl of the eluate was added to 20 μl of the PCR master mix.

DNA extraction directly from field sampling was carried out according to the manufacturer using the ChargeSwitch^® ^gDNA kit. The principle of this extraction method is the use of magnetic beads. At low pH, the magnetic beads have a positive charge that binds the negatively charged nucleic acid backbone of DNA. Proteins and other contaminants do not bind and are removed by washing. For the elution of the bound DNA, the charge of the magnetic beads was neutralised by raising the pH to 8.5 using a low salt elution buffer. The purified DNA was released into the elution buffer; the yield from 50 μl of blood was up to 2 μg. For PCR, 1 μl from the extract was used as the template.

### PCR

TBR-PCR was used in the current study for the detection of *T. brucei *s.l. The reaction is species specific, amplifying a 173 bp product from a repetitive satellite sequence of 177 bp size [44]. The copy number of the satellite sequence was reported to be 10,000 copies/genome [45] ensuring high sensitivity. The primer sequences used were TBR1-5'-CGA ATG AAT ATT AAA CAA TGC GCA GT-3' and TBR2-5'-AGA ACC ATT TAT TAG CTT TGT TGC-3' [44].

The reaction volume used was 25 μl and contained NH_4 _Buffer (Bioline, London, UK) which comprises 16.0 mM (NH_4_)_2_SO_4_, 67 mM Tris-HCl (pH 8.8 at 25°C) and 0.01% Tween 20. Moreover, 0.4 μM of each primer, 1.5 mM Mg^2+^, 200 μM of each of the four-deoxynucleoside triphosphates (dNTP) and one Unit of BIOTAQ RED DNA Polymersase (Bioline) were added. One positive control (genomic DNA) and one negative control were run with each PCR. The reaction conditions used were as follows: 1 cycle of 95°C for seven minutes followed by 30 cycles of 94°C for one minute, 55 °C for one minute and 72 °C for 30 seconds. The reaction was carried out in a DNA Engine DYADTM Peltier Thermal Cycler. Amplified products were visualized on a 1% (w/v) agarose gel electrophoresis.

### Data analysis

The difference in trypanosome detection between the different sample preparations was analysed using the Chi-squared test (the degrees of freedom were noted as a subscript to the χ^2 ^-statistic) and was computed using Minitab version 15 (Minitab, Inc.). Differences were considered to be significant at p < 0.05. For values containing expected frequencies of less than five, Fisher's exact test was used instead of the Chi-squared test. The Kappa value was used to determine the level of agreement between the diagnostic test and the gold standard. The Kappa value lies between 0 and 1, where the following definitions apply: 0.8-1, very good agreement between tests; 0.6-0.8, good agreement; 0.4-0.6, moderate agreement; 0.2-0.4, fair agreement; 0-0.2, poor agreement; 0, the association is no better than expected from chance alone, a negative Kappa value indicates that the two tests agree less than would be expected by chance [46,47].

## Competing interests

The authors declare that they have no competing interests.

## Authors' contributions

HAA carried out the study, the molecular approaches and participated in preparing the manuscript. ETM participated in preparing and editing the manuscript. GH participated in editing the manuscript. SCW designed the study supervised the molecular approaches and participated in preparing and editing the manuscript. KP designed the study supervised the molecular approaches and participated in preparing and editing the manuscript. All authors read and approved the final manuscript.
